# Identification of Soybean Varieties Using Hyperspectral Imaging Coupled with Convolutional Neural Network

**DOI:** 10.3390/s19194065

**Published:** 2019-09-20

**Authors:** Susu Zhu, Lei Zhou, Chu Zhang, Yidan Bao, Baohua Wu, Hangjian Chu, Yue Yu, Yong He, Lei Feng

**Affiliations:** 1 College of Biosystems Engineering and Food Science, Zhejiang University, Hangzhou 310058, China; sszhu@zju.edu.cn (S.Z.); zhoulei_17@zju.edu.cn (L.Z.); chuzh@zju.edu.cn (C.Z.); ydbao@zju.edu.cn (Y.B.); wubaohua@zju.edu.cn (B.W.); hjchu@zju.edu.cn (H.C.); yuyue0416@zju.edu.cn (Y.Y.); yhe@zju.edu.cn (Y.H.); 2 Key Laboratory of Spectroscopy Sensing, Ministry of Agriculture and Rural Affairs, Hangzhou 310058, China

**Keywords:** soybean, hyperspectral imaging technology, convolutional neural network, pixel-wise spectra, a majority vote

## Abstract

Soybean variety is connected to stress resistance ability, as well as nutritional and commercial value. Near-infrared hyperspectral imaging was applied to classify three varieties of soybeans (Zhonghuang37, Zhonghuang41, and Zhonghuang55). Pixel-wise spectra were extracted and preprocessed, and average spectra were also obtained. Convolutional neural networks (CNN) using the average spectra and pixel-wise spectra of different numbers of soybeans were built. Pixel-wise CNN models obtained good performance predicting pixel-wise spectra and average spectra. With the increase of soybean numbers, performances were improved, with the classification accuracy of each variety over 90%. Traditionally, the number of samples used for modeling is large. It is time-consuming and requires labor to obtain hyperspectral data from large batches of samples. To explore the possibility of achieving decent identification results with few samples, a majority vote was also applied to the pixel-wise CNN models to identify a single soybean variety. Prediction maps were obtained to present the classification results intuitively. Models using pixel-wise spectra of 60 soybeans showed equivalent performance to those using the average spectra of 810 soybeans, illustrating the possibility of discriminating soybean varieties using few samples by acquiring pixel-wise spectra.

## 1. Introduction

Soybean (*Glycine max (L.) Merrill*) is one of the most important agricultural products. It provides fat and protein for humans and livestock [1,2]. The discrimination of soybean seed attributes such as genotypes [3] and protein content [4] has become an important research orientation in agronomy in recent years. Different soybean varieties have different genetic purity, physical purity, germination ability, and vigor, which are related to the quality attributes, such as nutritional value, stress resistance ability, final yield, etc. [5,6,7]. The quality attributes of soybeans are the parameters that producers are most concerned about, and different soybean varieties have different quality attributes, which also make soybean variety identification an important issue in the research.

Genetic analysis and chemometrics-based technologies are known as powerful tools for the identification of accurate seed varieties [8,9,10,11]. However, these methods are only suitable for the laboratory environment, and adequate skills are also required for operational staff. What’s more, these methods are all destructive with high costs and low efficiency. The aftertreatment of the chemicals used in the testing process is also an important issue. Compared with other detection methods, manual visual identification is the most convenient method. However, it is time-consuming and requires experts with extensive experience and knowledge, and the accuracy cannot be guaranteed.

Soybean seeds of different varieties show some differences of external characteristics (including texture features, colors, damage, etc.), chemical composition, and other aspects. With the advantages of being non-chemical, highly efficient, and easy to operate, computer vision has been proposed as a rapid and non-destructive discriminant method for seed variety identification based on the differences of external characteristics [12,13]. Furthermore, it is easy to develop a portable device on the basis of computer vision and image processing algorithms, which can be arranged for outdoor environment applications. For different soybean varieties that have quite similar external characteristics, computer vision may have trouble with achieving satisfactory results. Apart from external attributes, differences of chemical compositions inside samples are also of significant importance for the identification of soybean seed varieties. Near-infrared spectroscopy has been widely used to identify seed varieties regarding the chemical composition differences among samples [14,15].

Hyperspectral imaging, integrating both the computer vision and near-infrared spectroscopy, is being currently studied with the advantages of the two techniques [16,17,18]. Hyperspectral imaging acquires internal and external features simultaneously. Nowadays, studies have used hyperspectral imaging to identify seed varieties [19,20]. Generally, near-infrared spectroscopy conducts point measurement, and it is quite difficult to obtain the spectral information of each region within the seed. Different from near-infrared spectroscopy, hyperspectral imaging can acquire the spectral information of samples at the pixel-wise level, providing the comprehensive spectral information of the sample. Pixel-wise spectra also contain the sample information and can be used for analysis. Studies have utilized both average spectra and pixel-wise spectra to build training models [21,22,23]. Compared with average spectra of samples, pixel-wise spectra contain more detailed information, and the number of pixel-wise spectra is much larger than the number of average spectra of samples. Using pixel-wise spectra for training not only contains more detailed information, but also increases the training number, especially when the number of sample is small. However, how to fully explore the spectral features of average spectra and pixel-wise spectra is challenging.

Deep learning has been proven as a strong method for feature learning. Features can be learned automatically and deeply by different deep learning architectures, and can be extracted as inputs of classification and regression models. Moreover, deep learning is quite efficient for big data analysis. Recent studies have proved the effectiveness of deep learning in spectral data analysis [19,24,25,26,27,28,29,30]. There are some researches that have applied deep learning methods in analyses of hyperspectral images in the agriculture field. Qiu et al. used convolutional neural network (CNN) to identify rice seed varieties based on hyperspectral images [19]. K-nearest neighbors (KNN) and support vector machine (SVM) models were also built for comparison. The results of the CNN model obtained better performances than the KNN and SVM models in most cases, which revealed the effectiveness of analyzing spectral data based on CNN. Yu et al. discriminated the freshness of shrimp during cold storage using visible and near-infrared hyperspectral imaging techniques combined with a deep learning algorithm [27]. A stacked auto-encoders (SAEs)-based deep learning algorithm was used to extract the features of two freshness grades (fresh and stale) of shrimps. Satisfactory results were achieved with classification accuracy reaching 96.55% and 93.97% for training and prediction sets, respectively. Yu et al. detected nitrogen (N) concentration in oilseed rape leaf using SAE and a fully-connected neural network (FNN) [30]. The SAE–FNN model obtained decent results with R^2^_P_ = 0.903, RMSEP (Root-Mean-Square Error of Prediction Set)= 0.307% and RPD_P_ (Residual Predictive Deviation of Prediction Set) = 3.238 for N concentration, indicating the possibility of N concentration detection in oilseed rape leaf by the combination of a hyperspectral imaging technique and deep learning method.

Most of the researchers would obtain large scales of data of samples to build classification models that could acquire decent results. However, it is time-consuming and laborious. Pixel-wise spectra could provide much larger data volumes than an average spectra of samples, and deep learning has great potential for pixel-wise spectral analysis. Thus, we explore the possibility of achieving decent discriminant results of soybean variety identification with few samples based on hyperspectral imaging combined with CNN in this study. Different numbers of soybeans were used to build models based on both average spectra and pixel-wise spectra. To further improve the performance of pixel-wise CNN models, a majority vote was also applied to identify a single soybean variety.

## 2. Materials and Methods

### 2.1. Sample Preparation

**Figure 1:**
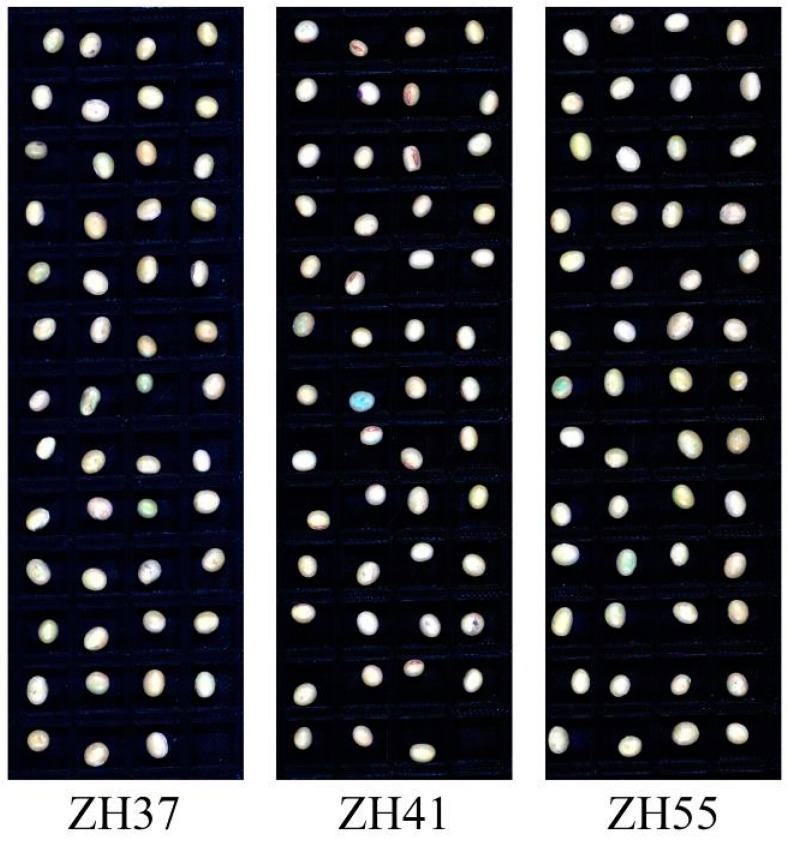
RGB (Red Green Blue) images of three varieties of soybeans.

Three varieties of soybeans, including Zhonghuang37 (ZH37), Zhonghuang41 (ZH41), and Zhonghuang55 (ZH55), were purchased from a local seed company in Changzhou, Hebei province, China. For each variety, 1890 intact and healthy soybeans were prepared. Single soybean kernels were placed separately in a black sampling plate for hyperspectral image acquisition (shown in Figure 1). Soybeans of these three varieties showed no significant visual differences. 

### 2.2. Hyperspectral Image Acquisition and Correction

In this study, a near-infrared hyperspectral imaging system covering the spectral range of 874–1734 nm was used to acquire hyperspectral images of soybeans. Table 1 presents the main components of the near-infrared hyperspectral imaging system.

Seeds were placed in the sampling plate separately (Figure 1). To acquire non-deformable and clear hyperspectral images, three deciding factors of image quality were adjusted for the system. The distance between the sampling plate and the camera lens, the speed of mobile platform, and the exposure time were set as 12.6 cm, 11 mm/s, and 3000 μs, respectively. The acquired hyperspectral images were raw images with light intensity, and needed to be corrected as the reflectance hyperspectral images. The image correction follows the equation:

(1) $$I_{c}=\frac{I_{r}-I_{d}}{I_{w}-I_{d}}$$

where *I_c_* is the corrected image, *I_r_* is the raw image, *I_w_* is the white reference image that is obtained by using a white Teflon board with a high reflectivity (nearly 100%), *I_d_* is the dark reference image for dark current removal by using a black plate with nearly 0% reflectivity along with turning off the light sources.

### 2.3. Spectral Data Extraction and Preprocessing

**Figure 2:**
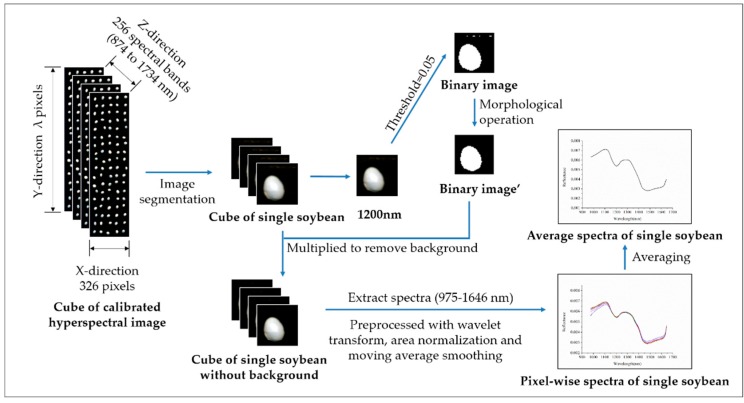
Procedures of spectral data extraction and preprocessing.

In this study, sample average spectra and pixel-wise spectra were both used. In this study, 200 wavebands ranging from 975 to 1646 nm of hyperspectral images were studied. Figure 2 shows the procedures of spectral data extraction and preprocessing. Firstly, single soybeans were segmented from the background. Gray-scale images at 1200 nm were used to form binary images (soybean regions as ‘1’ and background regions as ‘0’) to isolate the soybeans from the background of near-infrared hyperspectral images. Then, the binary images were multiplied with the gray-scale images at each wavelength to remove the background. Pixel-wise spectra within each soybean were preprocessed by wavelet transform [31] (wavelet function Daubechies 6 with decomposition level 3 for both spectral ranges) followed with an area normalization and a moving average smoothing (7 points). The equations of area normalization (2) and a moving average smoothing (3) are as follows:

(2) $$X_{i}=\frac{A_{i}}{\sum_{j=1}^{N}A_{j}}$$

(3) $$M_{i}=\frac{\sum_{k=i-3}^{i+3}X_{k}}{7}$$

where *A_i_* and *X_i_* are respectively the spectral values before and after normalization at the *i*^th^ wavelength; *N* is the number of spectral wavelengths; and *M_i_* is the spectral value after moving average smoothing with 7 points.

Pixel-wise spectra of each soybean were exacted and preprocessed, and the preprocessed spectra were then averaged to represent the soybean. Both pixel-wise spectra and the average spectrum of each soybean were studied. To specify the difference between pixel-wise spectra and average spectra, models using pixel-wise spectra were defined as pixel-wise models, and models using average spectra were defined as object-wise models.

### 2.4. Discrimination Models

#### 2.4.1. Deep Learning Methods

Deep convolutional neural network (DCNN) has become an emerging method for hyperspectra data analysis due to its strong ability for abstract feature learning [25]. In this research, a small-scale CNN architecture based on the model presented in literature [19] was designed, modified, and evaluated. In order to make full use of the advantages of big data provided by hyperspectral imaging technology, a new decision-making strategy was proposed. In addition, the strategy that separates different variety of seeds using the average spectra of each seed sample was used for comparison [19,32].

**Figure 3:**
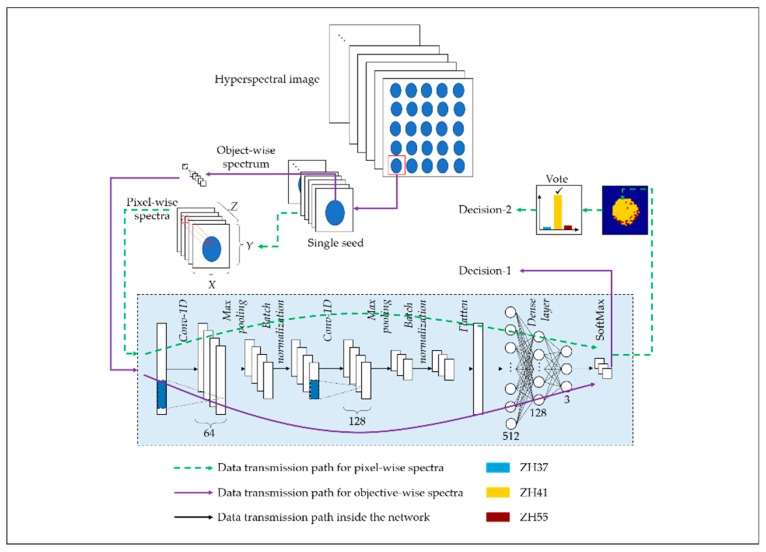
The block diagram of proposed majority vote strategy based on pixel-level spectra and conventional strategy based on mean spectra for a convolutional neural networks (CNN)-based classifier.

In this study, both pixel-wise spectra and average spectra were studied for soybean varieties identification. Figure 3 shows the CNN architecture and two different kinds of decision-making strategies. The CNN architecture is shown in the box with a background color of light blue in Figure 3. It consisted of two one-dimension convolution layers, each of which is followed by a Rectified Linear Units (ReLU) activation, a MaxPooling layer, and a batch-normalization process, forming a fully connected network constructed by three dense layers and a SoftMax layer. The function of the three parts could be summarized as follows:

(1) Convolution layer: Used for feature learning. The kernels in convolution layers are filters with the shape of 3*1. The weights of the kernels can be automatically fitted by training. The convolution layers can recognize the patterns in spectral curves such as peaks, slopes, minimums, etc., which is similar to corners and edges in images.
(2) Max pooling layer: The main features are screened out and the dimension of the feature map and calculation amount are also reduced. Thus, this layer is used to prevent over-fitting and improve the generalization ability of the model.
(3) Dense layers connected with SoftMax layer: A classifier trained to establish the relationship between the extracted feature map and the corresponding classification results.

The numbers of kernels in the convolution layers were 64 and 128 respectively, with a kernel size of 3, stride of 1, and padding of 0. The MaxPooling layers were set with a pool size of 2 and a stride of 2. The numbers of the neurons in the dense layers were defined as 512, 128, and 3, in that order. All the dense layers are activated by the ReLU function.

The training task was performed by minimizing the SoftMax Cross Entropy Loss using the stochastic gradient descent (SGD) algorithm. The learning rate was optimized and set as 0.005. The batch size was set as 1024 for pixel-level input and 200 for mean spectra input. The train epoch was defined as 300.

In the proposed decision-making strategy, each pixel in the spectral image of an individual soybean seed sample was fed into the CNN model and processed to output the classification result (Decision-1). After traversing each pixel in the spectral image, a map of the prediction results of each pixel could be obtained. A majority vote was employed to make the final decision for classification (Decision-2). In other words, the largest number of categories determines the final classification decision of a complete spectral image of a soybean seed sample. The data transmission flow for the strategy based on majority vote is marked using green dotted lines with an arrow, and that for the conventional strategy based on mean spectra is marked by a purple solid line with an arrow in Figure 3. 

#### 2.4.2. Principal Component Analysis

In order to explore the qualitative differences among three varieties of soybeans, principal component analysis (PCA) was applied to near-infrared spectra. As a multivariate statistical method, PCA is widely used for feature extraction and data dimension reduction by analyzing the correlation among variables. PCA transforms the raw data into linearly independent variables, which are called the principal components (PCs). The first few PCs contain most of the information of hyperspectral images, and could reveal some differences among the different varieties of samples. Therefore, the first three PCs obtained from the average spectra of the soybeans in the training set were applied to form the PCA scores scatter plots in this study [33].

## 3. Results and Discussion

### 3.1. Spectral Profiles

**Figure 4:**
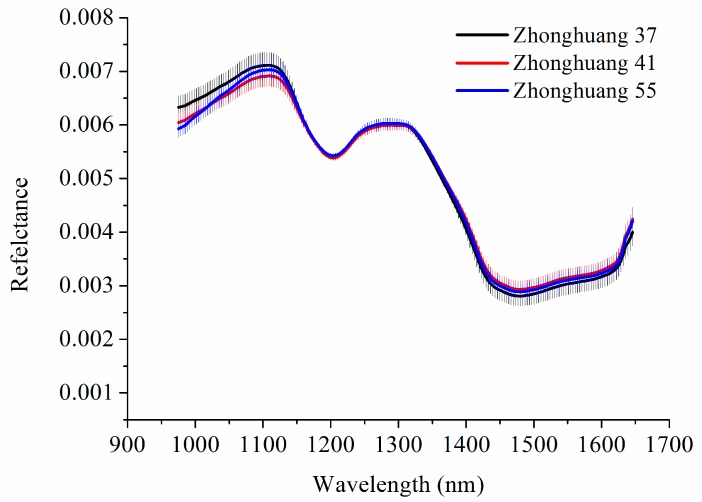
Average spectra with SD of three varieties of soybeans.

The near-infrared hyperspectral imaging system with the range of 874–1734 nm was used to obtain the reflectance spectra of three varieties of soybeans. Because of the noises existed at the beginning and end of the spectral range, the spectral range of 975-1646 nm was used for further data analysis. To reveal the differences among three varieties of soybeans, the average spectra with standard deviation (SD) of a training set of three varieties of soybeans were presented in Figure 4. The variation trend of the three spectral curves were similar. The peaks and valleys of the spectral curves were same, with peaks at 1109 and 1284 nm, and valleys at 1204 and 1456 nm. There were some differences in the spectra range of 975–1120 nm, while overlaps existed in the rest of the spectra.

### 3.2. PCA Scores Scatter Plot Analysis

**Figure 5:**
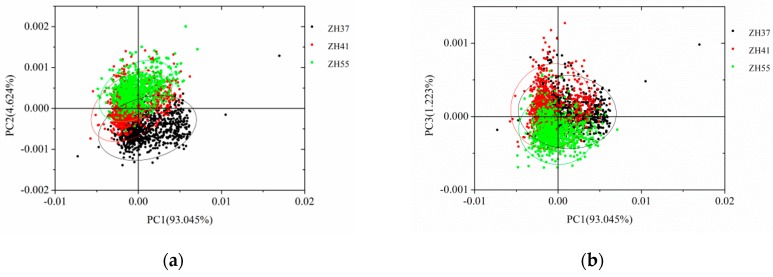

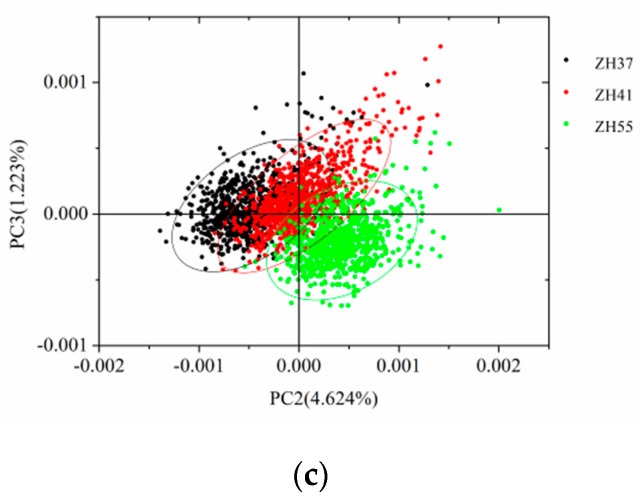
Scores scatter plot of soybeans (**a**) PC1 vs. PC2; (**b**) PC1 vs. PC3; (**c**) PC2 vs. PC3. PC: principal components.

PCA scores scatter plot analysis was applied in the spectra of three varieties of soybeans (Figure 5). For each soybean variety, the average spectra of 810 samples in the training set were used for PCA analysis. The first three PCs explained 98.892% of the samples’ variance (93.045% for PC1, 4.624% for PC2, and 1.223% for PC3). Thus, the first three PCs of soybeans were used to form the PCA scores scatter plot. From Figure 5, each variety was clustered together according their own attributes. The overlaps of three varieties of soybeans existed in all three images within Figure 5, while the scores scatter plot of PC2 versus PC3 showed the least overlap. Although some differences were revealed by the PCA scores scatter plot, further data processing still needed to enlarge the differences of three varieties of soybeans.

### 3.3. Classification Models on Average Spectra and Pixel-Wise Wavelengths

After spectral extraction and preprocessing, soybeans were divided into the training set, the validation set, and the prediction set for CNN models. In sum, 810 soybeans of each variety were used for training. The average spectra of 10, 20, 30, 60, 90, 180, 360, 540, 720, and 810 soybeans of each variety in the training set were used for training for average spectra modeling, and the pixel-wise spectra of 10, 20, 30, and 60 soybeans of each variety were used for pixel-wise spectra modeling. Another 180 soybeans of each variety were used for validation. The average spectra and pixel-wise spectra of the validation set were used to validate pixel-wise CNN models and object-wise CNN models, respectively. The parameters in CNN were chosen by an empirical method at beginning. Then, parameters were adjusted according to the accuracy of the validation set. According to the performance of the validation sets, all the CNN models obtained satisfactory results using the parameters selected in Figure 3. The remaining 900 soybeans of each variety were used for prediction. Object-wise models were used to predict the average spectra. Pixel-wise models were used to predict both average spectra and pixel-wise spectra. To build CNN models, the category values of ZH37, ZH41, and ZH55 were assigned as 0, 1, and 2, respectively.

Table 2 reveals the results of the object-wise CNN models that were used to predict the average spectra. For the training set, CNN models based on different sample numbers all achieved satisfactory results, with all accuracies reaching 100%, while the accuracy of the validation and prediction sets showed obvious differences. The number of samples used for training was small to some extent, which resulted in insufficient representativeness of the sample. Thus, over-fitting existed for the training sets. For the validation and prediction sets, the accuracy increased at first, and then became stable with the expansion of the sample size used for training. As the number of each soybean variety increased from 10 to 810, the accuracy of the validation set and prediction set increased from lower than 90% to above 99%. Zhao et al. explored the influence of different sample sizes used for maize variety training. Support vector machine (SVM) and radial basis function neural network (RBFNN) models built on the average spectra were developed [31]. Qiu et al. also studied the effect of classification accuracy introduced by different numbers of training samples (100, 200, 300, 400, 500, 600, 700, 800, 900, 1000, 1500, 2000, 2500, and 3000) on the basis of the average spectra [19]. The same changing tendency of the prediction set was observed in these two studies.

As for comparison, a simplified ResNet architecture [34] (with two 1-D convolution layers) and a simplified Inception architecture [25] (with two 1-D convolution layers and two parallel data channels) were used for modeling and evaluation based on objective-wise spectra (Table 3). In order to facilitate comparison, only 10 and 810 samples were selected.

Using 10 samples of each variety for training, the ResNet architecture achieved 100%, 61.111%, and 74.000% for the training set, validation set, and prediction set, respectively. The training time was 18.210 seconds. Considering 810 samples of each variety for training, the ResNet architecture achieved 100%, 93.333%, and 97.556% for the training set, validation set, and prediction set, respectively. The time for training was 190.509 seconds.

The Inception model trained by 10 samples of each variety achieved a slightly better result (100%, 74.630%, and 89.111%) than ResNet with a time consumption of 50.004 seconds for training, and the model built based on inception architecture using 810 samples of each variety achieved a similar result (100%, 96.852%, and 98.889%) to the ResNet model, with a time consumption of 95.604 seconds for training.

The performance of the simplified ResNet architecture and Inception architecture was worse than that of the simple CNN model. The complex architectures of these two models need much more parameters, which would lead to the over-fitting problem. The time consumption for modeling was also higher than that of the CNN model with simpler architecture, which was good enough for practical applications.

Compared with object-wise CNN models, CNN models using pixel-wise spectra for training, validation, and prediction were also studied (Table 4). In order to compare the modeling performance of different CNN models, the same pixels of validation and prediction sets were selected for CNN models using different soybean numbers for training, with 208,788 pixels prepared for training and 1,057,007 pixels chosen for prediction. The prediction set of pixel-wise spectra and average spectra were both presented in Table 4. 

For the training set, the accuracy was varied from 93%–96% for all four CNN models. There were no obvious accuracy differences of the pixel-wise validation and prediction sets for CNN models built using 10, 20, and 30 soybean kernels of each variety. CNN models using 60 kernels of each variety outperformed the other three models in pixel-wise spectra prediction and average spectra prediction, with accuracy reaching 83.875% and 95.556%, respectively. Comparing the prediction sets of pixel-wise spectra and average spectra, the prediction accuracy of average spectra was higher than that of pixel-wise spectra, with accuracy in the range of 79%–96% for the three varieties of soybeans. What is more, the accuracy of the prediction of average spectra reached 95.556% by CNN models using 60 soybeans for model building, which was close to the CNN models using the average spectra of 810 samples of each variety. These results illustrated the feasibility of using pixel-wise CNN models to identify soybean varieties. Zhang et al. also used pixel-wise spectra to build SVM models to predict the pixel-wise spectra and average spectra of coffee beans. They selected 2000 pixels of each variety randomly from 600,000 pixels for modeling, and decent results were also obtained [21]. Feng et al. also extracted pixel-wise spectra to build SVM, k-nearest neighbors algorithm (KNN), and RBFNN models for raisin variety classification. It also achieved good results [33]. These studies proved the great potential to establish models based on pixel-wise spectra to obtain satisfactory results.

Comparing the computation time of object-wise CNN models and pixel-wise CNN models, the computation time of both two models would increase as the number of samples used for the models increased. Although the modeling time of pixel-wise CNN models was longer than that of object-wise CNN models, the input variables of pixel-wise CNN models were a thousand times more than those of object-wise CNN models, so the computation time was still acceptable.

A majority vote was also applied to the pixel-wise CNN models to improve modeling performance. Pixel-wise prediction was conducted to each soybean kernel, and the specific variety of each soybean kernel was determined by calculating the percentage of pixel-wise prediction results of each variety in one single soybean, and assigned as the variety with the largest percentage. 

The results of pixel-wise CNN models and the vote results of each soybean variety are shown in Table 5. Compared with the pixel-wise prediction of each variety, variety identification by voting performed better among all the three sets. The results of the training set by voting all reached 100%, while the accuracy of the pixel-wise training set was in the range of 83%–99%. For the validation and prediction sets, the classification accuracy using pixel-wise spectra was inferior to that achieved by using voting for variety determination, and the differences became larger with the increased number of soybeans in the training set. With the increased number of soybeans in the training set, the prediction results were also improved for both pixel-wise prediction and voting. The prediction results of pixel-wise CNN models using 60 soybeans by voting performed better than that of the average spectra.

The overall results indicated that vote-based pixel-wise CNN models built on a few samples (60 kernels of each variety) could be used to identify soybean varieties, which significantly improved the sampling and modeling efficiency.

### 3.4. Prediction Maps

**Figure 6:**
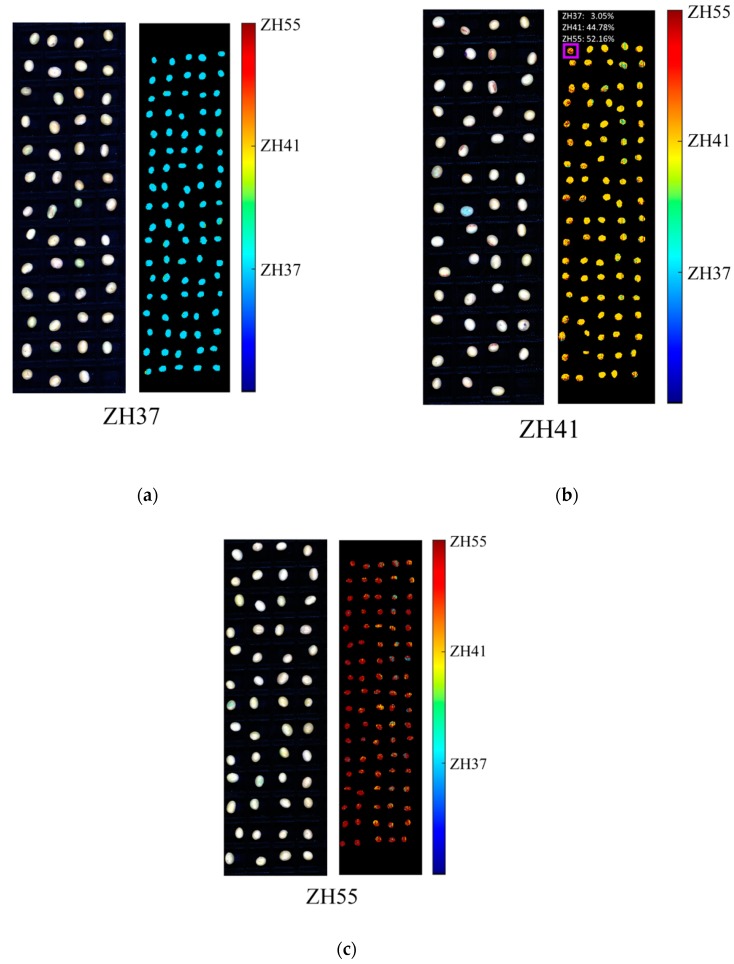
Pseudo-color images (1000, 1200, and 1400 nm) and prediction maps of a randomly selected image of each variety based on pixel-wise CNN models built with 60 soybeans of each variety: (**a**) ZH37, (**b**) ZH41, and (**c**) ZH55.

In order to present the classification results more intuitively, prediction maps were obtained by applying CNN models on the pixel-wise spectra of 60 soybean kernels of each variety. Figure 6 reveals the pseudo-color images (1000, 1200, and 1400 nm) of near-infrared spectra and the corresponding pixel-wise prediction maps of three varieties of soybeans. One near-infrared spectral image of each variety was randomly selected to form the prediction map. Distinguishable prediction differences could be observed from Figure 6. The color distribution indicates the prediction result of the variety of soybean in the near-infrared spectral image. Blue, yellow, and orange colors stand for the prediction results assigned to ZH37, ZH41, and ZH55, respectively. It can be seen from Figure 6b,c that the color differences could be found within a single soybean kernel. Satisfactory results were obtained since nearly all the soybean kernels were correctly classified. Specially, the marked kernel in Figure 6b was the only one being misclassified as ZH55, because in this situation, 52.16% of pixels were identified as ZH55. On the whole, the prediction maps revealed the possibility of classifying soybean variety by calculating the ratio of classification results of pixel-wise CNN models built on a few samples.

## 4. Conclusions

Hyperspectral imaging coupled with a CNN model showed great potential for soybean variety classification. Three varieties of soybeans (Zhonghuang37, Zhonghuang41, and Zhonghuang55) were prepared for study to explore the possibility of building reliable models with few samples. Pixel-wise spectra and average spectra were both extracted for comparison. PCA was applied to form PCA scores scatter plot images for qualitative analysis, which presented the differences of three varieties of soybeans. Object-wise and pixel-wise CNN models were both established. Average spectra were used for prediction in both object-wise CNN models and pixel-wise CNN models. Pixel-wise spectra were only used for prediction in pixel-wise CNN models. Object-wise CNN models achieved decent results when the samples used for training had more than 360 kernels of each variety, while satisfactory results were obtained by pixel-wise CNN models using a few samples (60 kernels of each variety). A majority vote, which further improved the classification accuracy, was adopted to make the final decision for variety determination. CNN models based on pixel-wise spectra with a few samples achieved good results. The results illustrated the great potential and advantage of hyperspectral imaging to identify soybean varieties with a few samples using pixel-wise spectra rather than a large scale of samples using average spectra. The pixel-wise models performed well on average spectra, extending the use of pixel-wise spectra. Moreover, CNN could deal with a large number of pixel-wise spectra efficiently and rapidly, illustrating the great potential of CNN in related studies. The results in this study would also help to identify other samples with a similar situation. In future research, samples of different origins, years, and varieties should be taken into consideration.

## Figures and Tables

**Table 1:** Main components of the near-infrared hyperspectral imaging system.

Component	Near-Infrared Hyperspectral Imaging System
Imaging spectrograph	ImSpector N17E (Spectral Imaging Ltd., Oulu, Finland)
Camera	InGaAs camera (Xeva 992; Xenics Infrared Solutions, Leuven, Belgium)
Lens	OLES22 (Spectral Imaging Ltd., Oulu, Finland)
Image size (Image width × image length × wavebands)	326 × λ × 256
Acquisition mode	Line-scan
Light sources	3900 Lightsource (Illumination Technologies Inc., Syracuse, New York, USA)
Mobile platform	IRCP0076 electric displacement table (Isuzu Optics Corp., Taiwan)

**Table 2:** Object-wise CNN models used to predict average spectra.

Number ^1^	Accuracy (%)			Computation Time ^5^ (s)
	Tra-average ^2^	Val-average ^3^	Pre-average ^4^	
**10**	100	75.556	87.296	5.742
20	100	79.259	88.593	6.103
30	100	85.370	93.370	6.490
60	100	90.370	96.333	7.016
90	100	94.074	97.778	11.590
180	100	95.185	98.222	15.862
360	100	98.333	99.259	21.662
540	100	99.074	99.296	28.260
720	100	99.259	99.481	35.667
810	100	99.444	99.778	38.935

^1^ The soybean number of each variety used in the training set; ^2^ Accuracy of training set based on object-wise CNN models; ^3^ Accuracy of object-wise CNN models to validate average spectra; ^4^ Accuracy of object-wise CNN models to predict average spectra; ^5^ Computation time of training set.

**Table 3:** Object-wise ResNet and Inception models used to predict average spectra.

Model	Number ^1^	Accuracy (%)			Computation Time ^5^ (s)
		Tra-average ^2^	Val-average ^3^	Pre-average ^4^	
**ResNet**	**10**	100	61.111	74.000	18.210
	810	100	93.333	97.556	190.509
Inception	10	100	74.630	89.111	50.004
	810	100	96.852	98.889	95.604

^1^ The soybean number of each variety used in the training set; ^2^ Accuracy of training set based on object-wise CNN models; ^3^ Accuracy of object-wise CNN models to validate average spectra; ^4^ Accuracy of object-wise CNN models to predict average spectra; ^5^ Computation time of the training set.

**Table 4:** Pixel-wise CNN models used to predict pixel-wise spectra and average spectra.

Number ^1.^	Pixels ^2^			Accuracy (%)				Computaion Time ^7^ (s)
	Training	Validation	Prediction	Tra-pixel ^3^	Val-pixel ^4^	Pre-pixel ^5^	Pre-average ^6^	
**10**	12,546	208,788	1,057,007	94.165	73.002	74.741	79.741	315
20	24,719			93.337	73.632	74.736	80.370	468
30	37,280			94.612	76.102	77.864	88.556	558
60	75,969			92.069	81.725	83.875	95.556	1140

^1^ The soybean number of each variety used in the training set; ^2^ Pixels used for training, validation, and prediction of pixel-wise models; ^3^ Accuracy of training set based on pixel-wise CNN models; ^4^ Accuracy of pixel-wise CNN models to validate pixel-wise spectra; ^5^ Accuracy of pixel-wise CNN models to predict pixel-wise spectra; ^6^ Accuracy of pixel-wise CNN models to predict average spectra; ^7^ Computation time of the training set.

**Table 5:** The results of pixel-wise CNN models and the vote results of each soybean variety. ZH37: Zhonghuang37, ZH41: Zhonghuang41, and ZH55: Zhonghuang55.

Set	Number ^1^	Accuracy (%)			
		ZH37	ZH41	ZH55	All
Tra-pixel **^2^**	10	99.604	94.138	88.035	94.165
	20	99.429	92.553	87.214	93.337
	30	98.989	94.390	89.876	94.612
	60	97.056	83.849	94.552	92.069
Val-pixel **^3^**	10	91.677	76.386	49.875	73.002
	20	90.519	79.339	50.071	73.632
	30	86.811	82.075	58.803	76.102
	60	82.521	78.030	84.583	81.725
Pre-pixel **^4^**	10	94.938	77.774	51.766	74.741
	20	94.703	78.737	51.069	74.736
	30	92.277	82.186	59.416	77.864
	60	87.901	79.681	83.859	83.875
Tra-vote **^5^**	10	100	100	100	100
	20	100	100	100	100
	30	100	100	100	100
	60	100	100	100	100
Val-vote **^6^**	10	100	96.111	58.333	84.815
	20	100	98.333	58.333	85.556
	30	100	98.889	72.222	90.370
	60	99.444	96.111	98.889	98.148
Pre-vote **^7^**	10	100	96.111	57.889	84.667
	20	100	97.000	57.556	84.852
	30	100	99.222	74.444	91.222
	60	100	96.667	99.667	98.778

^1^ The soybean number of each variety used in the training set; ^2^ Training set based on pixel-wise CNN models used to predict pixel-wise spectra; ^3^ Validation set of pixel-wise CNN models used to predict pixel-wise spectra; ^4^ Prediction set of pixel-wise CNN models used to predict pixel-wise spectra; ^5^ Training set of pixel-wise CNN models used to vote the percentage of pixel-wise prediction results; ^6^ Validation set of pixel-wise CNN models used to vote the percentage of pixel-wise prediction results; ^7^ Prediction set of pixel-wise CNN models used to vote the percentage of pixel-wise prediction results.
